# Studies in Mice Reveal a Role for Anthrax Toxin Receptors in Matrix Metalloproteinase Function and Extracellular Matrix Homeostasis

**DOI:** 10.3390/toxins5020315

**Published:** 2013-02-06

**Authors:** Claire Reeves, Pelisa Charles-Horvath, Jan Kitajewski

**Affiliations:** 1 Department of Ob/Gyn, Columbia University Medical Center, New York, NY 10032, USA; E-Mails: creeves2002@gmail.com (C.R.); pcc2104@columbia.edu (P.C.-H.); 2 Department of Pharmacology, Columbia University Medical Center, New York, NY 10032, USA; 3 Department of Pathology, Columbia University Medical Center, New York, NY 10032, USA; 4 Herbert Irving Comprehensive Cancer Center, Columbia University Medical Center, New York, NY 10032, USA

**Keywords:** anthrax toxin receptors, MT1-MMP, extracellular matrix homeostasis, anthrax intoxication

## Abstract

The genes encoding Anthrax Toxin Receptors (ANTXRs) were originally identified based on expression in endothelial cells suggesting a role in angiogenesis. The focus of this review is to discuss what has been learned about the physiological roles of these receptors through evaluation of the *Antxr* knockout mouse phenotypes. Mice mutant in *Antxr* genes have defects in extracellular matrix homeostasis. We discuss how knowledge of physiological ANTXR function relates to what is already known about anthrax intoxication.

## 1. Introduction

Anthrax Toxin Receptor 1 (ANTXR1) and Anthrax Toxin Receptor 2 (ANTXR2) are the human proteins that serve as receptors for anthrax toxin [1,2], a discovery made ten years ago. Since that time, there has been an intense research effort to tell the story of how toxin interaction with these receptors leads to anthrax intoxication (reviewed in [3,4,5]). Although the ANTXR proteins evolved for a physiological function, they were eventually hijacked to facilitate entry of a lethal agent produced by the pathogen, *Bacillus anthracis*. In this review, we summarize recent reports on the normal function of anthrax receptors. We discuss studies that uncover the physiological function of ANTXR1 and ANTXR2, with a focus on analysis of the *Antxr1* and *Antxr2* knockout mouse phenotypes.

## 2. Discovery of the *ANTXR* Genes

**Figure 1:**
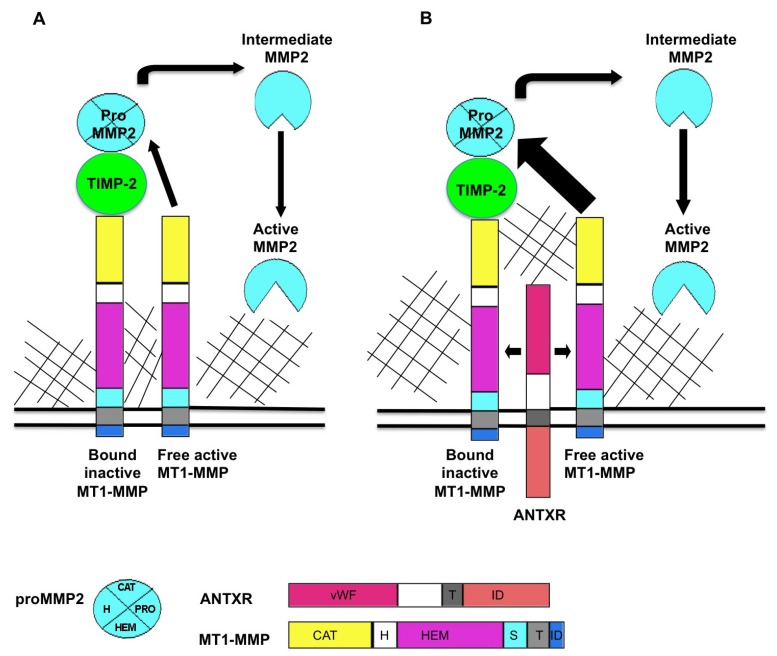
Proposed model for ANTXR regulation of MT1-MMP activity. MT1-MMP resides at the cell surface in its active form. MT1-MMP cleaves a variety of substrates including multiple ECM components. MT1-MMP also plays a role in MMP activation cascades, primarily in activation of pro MMP2. (**A**) The current accepted model for the generation of active MMP2 is that a ternary complex consisting of MT1-MMP/TIMP-2/pro MMP2 is cleaved by an adjacent active molecule of MT1-MMP to produce an intermediate form of MMP2. Fully active MMP2 is generated through *trans* activation by another active MMP2 protein; (**B**) ANTXRs bind putative ECM ligands including collagens and laminin. When ANTXRs and MT1-MMP are co-expressed, there is increased activation of MMP2, observed as decreased levels of pro MMP2. We propose that ANTXRs may interact with ECM components and facilitate multimerization and activation of ANTXR/MT1-MMP complex. ANTXRs may regulate MMP activity by (1) increasing MT1-MMP multimerization which, in turn increases pro MMP2 processing and by (2) mediating substrate specificity for MMPs. Pro: pro domain, vWF: von Willebrand factor, T: transmembrane domain, ID: intracellular domain, CAT: catalytic domain, H: hinge domain, HEM: hemopexin domain, S: stalk domain.

At the turn of the 21st century, targeting angiogenesis, the process by which new blood vessels sprout from pre-existing blood vessels, was recognized as an attractive therapeutic strategy for treating numerous pathological processes such as tumor growth, atherosclerosis and diabetic retinopathy. To that end, researchers performing discovery-based screens in either normal or disease associated endothelium identified novel proteins with putative roles in angiogenesis. One such screen led to the discovery of tumor endothelium marker 8 (*TEM8*), a gene that was found to be overexpressed in colorectal tumor endothelium compared to normal endothelium [6]. Similarly, capillary morphogenesis gene 2 (*CMG2*) was identified based on dramatically up-regulated expression in cultured human venous endothelial cells induced to undergo capillary morphogenesis *in vitro* [7]. Alternate splicing of the *TEM8* and *CMG2* genes result in multiple protein variants [1,2,6,7]. These splice variants have the potential to produce either membrane spanning (Figure 1) or secreted variants of TEM8 and CMG2, although new variants continue to be uncovered [8]. A detailed description of these variants is discussed in the literature (reviewed in [9]).

Shortly after the discovery of the *TEM8* and *CMG2* genes, separate searches for the host receptor(s) for anthrax toxin led to the discovery that the proteins encoded by *TEM8* and *CMG2* functioned at the cell surface as anthrax toxin receptors [1,2]. Hence the official NCBI gene designations for the *TEM8* and *CMG2* genes are *ANTXR1* and *ANTXR2*, respectively. We will use the official NCBI nomenclature when referring to the genes and to the proteins derived from these genes; a designation that we advocate for use to help maintain consistency in the field.

## 3. Studies Reveal a Role for ANTXRs as Regulators of Angiogenesis and Cell-Matrix Interactions

The original hypotheses regarding physiological Anthrax Toxin Receptor function in angiogenesis were based on expression in the endothelium. Consequently, many groups explored ANTXR function in endothelial cells. ANTXR1 has been demonstrated to be important for endothelial cell migration and capillary-like network formation [10,11]. Similarly, ANTXR2 is required for angiogenic processes such as endothelial proliferation and capillary-like network formation *in vitro* [12]. Given that the ANTXRs share structural similarities with α-integrin subunits, several groups also investigated whether extracellular matrix (ECM) proteins might serve as endogenous ligands for the ANTXRs. The extracellular domain of ANTXR1 has been shown to interact with collagen type I, the α3 subunit of collagen type VI, and gelatin [10,13]. Similarly, the extracellular domain of ANTXR2 has been shown to interact with collagen type IV and laminin [7]. Fibroblasts expressing mutant ANTXR2 protein exhibited deficient interactions with laminin, but not with collagen types I or IV [14]. Each of these studies took an approach that targeted specific ECM components when characterizing ANTXR/ECM interactions. Future studies evaluating interactions between each ANTXR and a wide array of ECM proteins will help determine whether ANTXRs exhibit specificity or promiscuity in their ligand binding abilities. It is also of interest that like integrins, ANTXR1 was shown to mediate cell spreading through association with the actin cytoskeleton [15]. Further evidence that the ANTXRs possess integrin-like qualities comes from a recent report where ANTXR1 was demonstrated to colocalize with alpha-smooth muscle actin at the cell surface. Co-immunoprecipitation experiments confirmed that the two proteins interact [16]. Exploration of antibodies that detect either masked or unmasked forms of ANTXR1 led to the hypothesis that ANTXR1, like integrins, exists in either an open or closed confirmation that is dependent upon receptor binding to the actin cytoskeleton [16]. Support for this hypothesis comes from recent studies demonstrating that anthrax toxin may take advantage of ANTXR/cytoskeletal interactions to process PA and that the cytoskeleton, in turn, is a target of anthrax toxin (reviewed in [17]). Collectively, these reports provide evidence that ANTXRs function as important regulators of cell-matrix interactions underlying various biological processes.

## 4. *Antxr1* Knockout Mice Demonstrate a Role for Antxr1 in Tumor Endothelium and Beyond

To elucidate ANTXR1 function, two independent groups generated *Antxr1* knockout (KO) mice [18,19]. The Leppla group targeted exon 13 of the *Antxr1* gene for deletion [18]. Exon 13 encodes the transmembrane domain of the Antxr1 protein. This targeting strategy would result in a protein consisting of the extracellular domain of Antxr1, which can be detected in the mutant mice [18]. These mice have likely lost normal Antxr1 function, but may retain some activities associated with the extracellular domain of Antxr1. Thus, we will refer to these mice as *Antxr1* mutant mice. The *Antxr1* mutant mice were used to evaluate receptor-mediated anthrax pathogenesis, and provided definitive evidence that gene deficiency affected anthrax intoxication. Beyond a role in intoxication, they noted that *Antxr1* mutant mice developed progressively misaligned incisors, and that female *Antxr1* mutant mice could get pregnant but failed to support normal embryonic development [18]. Thus, they established that diverse phenotypes arise from *Antxr1* deficiency. 

The St. Croix group targeted exon 1 of the *Antxr1* gene for deletion, which encodes part of the *Antxr1* promoter, the start codon and the signal peptide [19]. This targeting strategy would not be predicted to make Antxr1 protein and we will refer to these mice as *Antxr1* KO mice. Heterozygous intercrosses yielded *Antxr1* KO mice at the expected Mendelian ratios demonstrating that Antxr1 is dispensable for embryonic development. The researchers confirmed that Antxr1 transcripts and protein were not being expressed in the *Antxr1* KO mice by implanting tumors in wild-type (WT) and KO mice and then isolating tumor endothelial cells for RT-PCR and Western blot analysis. This approach was selected since Antxr1 is highly expressed in tumor endothelial cells. Although endothelial Antxr1 expression had been demonstrated to be important for angiogenic processes *in vitro*, evaluation of physiological and pathological angiogenic processes did not reveal overt angiogenic defects in *Antxr1* KO mice. With regards to physiological angiogenesis, there were no significant differences observed in the vasculature from *Antxr1* WT and KO mouse excisional wounds analyzed 7 days post wounding, nor were there alterations in the developing retinal vasculature when analyzed on postnatal days 3.5 through 11.5. Pathological angiogenesis in the *Antxr1* KO mice also appeared to be unaltered, when analyzed by performing syngeneic tumor implant studies. While there was significant growth inhibition of B16 melanoma tumors implanted in *Antxr1* KO mice, the growth delay did not correlate with significant differences in tumor vessel density or pericyte coverage. There were no detectable differences between WT and KO tumors when evaluating tumor endothelial cell apoptosis, tumor endothelial cell proliferation, tumor hypoxia, or tumor macrophage content. Thus, the mechanism by which Antxr1 contributes to tumor growth in this particular study was not delineated but clearly involved a stromal component of the tumor microenvironment. A recent follow-up study using these *Antxr1* KO mice bred to an immunodeficient background revealed slower growth of multiple human tumor xenografts that was due in part to reduced tumor vasculature [20,21]. Since the ANTXRs are highly expressed on tumor vasculature, it is logical to hypothesize that targeting the receptors might serve as a potential new strategy for developing anti-angiogenic therapies to treat cancer. Numerous studies addressing this hypothesis have been published and will not be elaborated upon in this review, as this topic is amply covered in the literature [9,21]. 

As *Antxr1* KO mice develop normally and thrive but fail to support strong tumor growth, it has been hypothesized that a key function of ANXTR1 may be to serve in stress-mediated responses [21]. This concept fits with the observation that TEM8 is highly expressed in endothelial cells in the stressful environment of a growing tumor. In fact, Chaudhary and St. Croix found that TEM8 is induced in cultured endothelial cells upon growth factor deprivation [20] or amino acid starvation [21], two conditions that tumor endothelial cells may be exposed to.

The predominant phenotype documented in the *Antxr1* KO mice was excessive deposition of extracellular matrix proteins, such as type I collagen and type VI collagen [19]. This ECM homeostasis defect was seen in numerous organs including the ovaries, uterus and skin and was documented in *Antxr1* KO mice that were 6 to 8 months old. The *Antxr1* KO mice displayed progressive misalignment of the incisors, similar to what had been reported for the *Antxr1* mutant mice [18,19]. While female *Antxr1* mutant mice displayed a fertility defect [18], male and female *Antxr1* KO mice were reported to be fertile when mated at 5 weeks of age and when analyzing litters born within the first 2 months of breeding [19]. Thus, it remains to be determined if older female *Antxr1* KO mice exhibit fertility defects. 

While human ANTXR1 was originally reported to be a tumor endothelial marker, the *Antxr1* KO mouse phenotype suggested more widespread expression of Antxr1, as ECM homeostasis defects are seen in tissues and cell types beyond tumor endothelium. Other studies support this supposition, demonstrating a more widespread pattern of Antxr1 expression at both the transcript and protein level in a variety of tissues including the skin, lung, intestine, brain, heart, kidney, spleen, liver, adrenal gland, thymus, muscle, pancreas, ovary and testis [8,18,22]. The *Antxr1* KO phenotype also supports the inclusion of uterus, incisors, bladder, stomach and tongue among the organs expressing Antxr1 [19]. Thus, ANTXR1 is highly expressed in tumor endothelium, functions in tumor growth, and appears to regulate ECM homeostasis in several organs of the mouse.

## 5. *Antxr2* Knockout Mice Exhibit Defective Parturition and Aberrant ECM Remodeling in the Reproductive Tract

The Kitajewski group generated *Antxr2* KO mice by targeting exon 1 for deletion [23]. *Antxr2* is not essential for normal development as heterozygous intercrosses yielded *Antxr2* KO mice in the expected Mendelian ratios. However, female *Antxr2* KO mice exhibited reproductive defects that varied with age: young mice exhibited a parturition defect while older mice were infertile. 

When mated at 6 weeks of age, *Antxr2* KO mice became impregnated but could not give birth on the expected due date. Analysis of the pregnant *Antxr2* KO reproductive tract near term revealed loss of the circular and longitudinal myometrial cell layers, the cells that produce uterine contractions. There was increased collagen deposition in what appeared to have been the myometrial layers. The *Antxr2* KO cervical stroma was collagen dense, indicating defective cervical remodeling. Thus, the block in the ability to give birth was due to defects in two processes required for successful parturition: coordinated uterine contractions and cervical ripening [23]. 

Older sexually mature *Antxr2* KO female mice, aged 2 to 6 months, would either miscarry their litters or exhibit parturition defects, as described above. *Antxr2* KO mice aged 7 months and beyond were not able to get pregnant. These age related changes in fertility suggested defects in the *Antxr2* KO reproductive tracts in the non-pregnant state upon aging. Reproductive tracts from 1-month-old prepubescent WT and *Antxr2* KO mice appeared similar. Reproductive tracts isolated from aged, sexually mature nulliparous *Antxr2* KO mice had a shortened, thickened shape in comparison to the thin, elongated reproductive tracts from WT animals. Histological analysis of the uterus and cervix showed aberrant deposition of ECM proteins including type I collagen, type VI collagen and fibronectin. The aberrant ECM deposition was progressive with age and caused severe disorganization of the cellular composition of these tissues such that normal uterine architecture was destroyed [23]. 

A subsequent report of *Antxr2* mutant mice, generated by targeting exon 12 for deletion, also describes a reproductive phenotype due to mutation of the *Antxr2* gene [24]. This targeting strategy produces an Antxr2 protein consisting of the extracellular domain [18] and this secreted Antxr2 variant could still have functional relevance. The parturition defect in the *Antxr2* mutant mice was similar to the defect described above, highlighting that either complete loss of Antxr2 protein [23] or generation of a protein lacking the transmembrane domain [24] results in parturition defects in mice. 

Thus, the *Antxr2* deficient phenotypes confirmed findings from analysis of *Antxr1* mutant mice [19]: the ANTXRs function in ECM homeostasis. However, the spectrum of tissues displaying ECM homeostasis defects differed for *Antxr1* mutant mice and *Antxr2* mutant mice. Unlike what had been observed for *Antxr1* KO mice, the ovaries from 6-month-old *Antxr2* KO mice did not reveal overt changes in ECM content that might interfere with follicular maturation or oocyte production and release [19,23]. Thus, impaired fertility in older *Antxr2* KO mice likely resulted from defects in uterine receptivity. 

## 6. *Antxr* Knockout Studies Suggest Functionally Redundant Roles for ANTXR1 and ANTXR2 in ECM Remodeling

There is likely to be overlap in Antxr1 and Antxr2 expression and function in light of the fact that mutant mice in either of these genes display an ECM homeostasis defect. Like Antxr1, expression of Antxr2 mRNA and protein is found in many organs including kidney, liver, lung, placenta, spleen, skeletal muscle, heart, skin, intestine, mammary gland and uterus [2,12,23]. Based on studies in mice, both receptors are expressed in lung, intestine, skin and uterus [12,19,22,23]. No co-expression studies have been published to date and this careful comparison may require the use of specific antibodies that recognize one receptor or the other, and which exhibit no cross reactivity. If one focuses on tissues that are known to require both Antxrs for normal function, loss of either *Antxr1* or *Antxr2* led to aberrant ECM accumulation in the uterus [19,23,24]. The increased ECM deposition in the uterus of *Antxr1* KO mice or *Antx2* KO mice suggests that both Antxrs function as regulators of ECM remodeling in the uterus. In *Antxr1* KO mice, there was also aberrant ECM deposition in the skin and intestine [19]. However, it is currently unknown if a similar phenotype is observed in *Antxr2* KO mice. Future studies with *Antxr1*; *Antxr2* double KO mice will help to elucidate if ANTXRs are fully or partially functionally redundant.

## 7. The ANTXRs Regulate MT1-MMP Activity

The ECM remodeling defects in the *Antxr2* KO suggested a defect in MMP activity in the mutant mice [23]. Gelatin zymography, used to evaluate the activity of MMP2 and MMP9, revealed reduced levels of active MMP2 in conditioned medium from *Antxr2* KO mouse embryonic fibroblasts (MEFs) when compared to WT MEFs. This observation was extended to the reproductive tract by assessing MMP2 processing in uterine lysates from *Antxr2* WT and KO mice. Increased levels of pro MMP2 and reduced levels of active MMP2 were found in the *Antxr2* KO uterine lysates when compared to WT lysates. The intermediate form of MMP2 was not detected in the *Antxr2* KO lysates but was detected in WT lysates. These experiments suggested that MMP2 activation, a process mediated by MT1-MMP, was defective in the *Antxr2* KO female mice [23].

MMP2 is a member of the matrix metalloproteinase (MMP) family, a group of zinc containing endoproteases that degrade ECM components that are grouped by protein structure and substrate specificity. MMP2 is known to degrade a variety of substrates including gelatin, collagens, fibronectin, laminin (reviewed in [25]). Pro-MMP2 is secreted as a zymogen and is activated at the cell surface by MT1-MMP and TIMP-2 [25,26,27,28]. MT1-MMP has a transmembrane and cytoplasmic domain, and is found on the cell surface in its active form. MT1-MMP is capable of activating other MMPs via a proteolytic mechanism, and it also cleaves a variety of ECM substrates ( [27] and reviewed in [25,29,30]). TIMP-2 is part of the family of secreted, homologous tissue inhibitors of metalloproteinases (TIMPs) [25,29,30]. As their name suggests, TIMPs were originally thought to inhibit MMP activity, but further studies showed that they also facilitate MMP activation in a context specific manner. TIMP-2 is commonly described as the endogenous inhibitor of MT1-MMP because high concentrations of TIMP-2 inhibit all MT1-MMP proteolytic activity. However, low concentrations of TIMP-2 facilitate formation of a TIMP-2/MT1-MMP/pro-MMP2 ternary complex as described below [25,31]. 

The classic model of pro-MMP2 activation is well studied (reviewed in [25]) and schematized in Figure 1A. MT1-MMP localized in the plasma membrane, binds to the N terminus of TIMP-2 to form a receptor for pro-MMP2. An adjacent TIMP-2-free MT1-MMP cleaves the pro MMP2 in the TIMP-2/MT1-MMP/pro-MMP2 complex to release an intermediate form of MMP2. An autocatalytic event then produces the fully activated form of MMP2 [26,32]. 

Our published report [23] suggested that the ANTXRs might be involved in this MMP2 activation complex, and one may propose that ANTXRs carry out this function via an association with MT-MMPs, as schematized in Figure 1B. Investigation of this hypothesis led to the discovery that both ANTXR1 and ANTXR2 positively regulate MT1-MMP activity in cultured cells [23]. Gelatin zymography on cultured cell media revealed that co-expression of MT1-MMP and either ANTXR1 or ANTXR2 consistently led to increased levels of MMP2 activation, as compared to cells expressing MT1-MMP alone. Moreover, the Mt1-mmp and Antxr2 proteins co-localize on the cell surface of MEFs and co-immunoprecipitation experiments confirmed that MT1-MMP and ANTXR proteins are found in a complex [23].

To initiate a discussion on how these proteins work, we propose that interactions between ANTXR and ECM components may promote multimerization and activation of a pericellular ANTXR/MT1-MMP complex (see Figure 1B). This hypothesis posits that the fibrosis present in various organs of *Antxr1* KO mice and both the pregnant and nonpregnant uterus and cervix of *Antxr2* KO mice are the result of reduced Mt1-mmp and Mmp2 proteolytic activity. This working model will depend on validation by further biochemical assessments, now facilitated by access to Antxr-deficient cell lines [19,23]. ECM homeostasis is a complex process that likely derives from a variety of enzymatic influences. Among those influences, we must include ANTXR1 and ANTXR2. Future investigation of this exciting topic will help our understanding of anthrax intoxication, fibrosis, and normal tissue physiology.

## 8. Are the Physiological Functions of Anthrax Toxin Receptors Important for Anthrax Intoxication?

Anthrax intoxication has been well studied, though questions surrounding the process still remain (reviewed in [3,4,17,33]) During anthrax intoxication, protective antigen (PA) binds to ANTXRs on the surface of target cells and mediates delivery of lethal factor (LF) and edema factor (EF) into the cytosol. Upon ANTXR binding, PA is cleaved by furin, a protease that has low expression in many cell types, and that is anchored to the golgi apparatus and plasma membranes [34,35]. While previous studies have established that furin cleaves PA, there is currently no data to indicate whether PA recruits furin on its own or if it takes advantage of the ANTXR’s ability to do so. Furin mediated cleavage of PA results in two PA fragments, PA63 and PA20. The PA63 fragment stays bound to the ANTXR and self associates to form a heptameric precursor pore. This “prepore” binds up to three molecules of lethal factor (LF) and edema factor (EF) and translocates into the cytoplasm of target cells. There, EF promotes the formation of cAMP, and LF proteolytically inactivates MAPKKs (reviewed in [3,4,17,33]). In the model of anthrax intoxication, ANTXRs are mostly regarded as the means of translocating the toxin into target cell. Mice lacking full function of Antxr1, Antxr2, or double mutants have been used to evaluate receptor function in mediating lethality due to toxin exposure *in vivo* [36]. These studies have established ANTXR2 as the primary anthrax toxin receptor *in vivo*, with ANTXR1 relegated to a minor role [36].

As discussed in this review, ANTXRs function as regulators of MT1-MMP activity, cell-matrix interactions and ECM remodeling. Could ANTXR regulation of MT1-MMP activity be relevant during anthrax pathogenesis? Using *in vitro* cell-free system and cultured human cancer cells, PA was identified as a substrate of MT1-MMP [37]. MT1-MMP is a protease that is activated by furin [38], and like furin, MT1-MMP exhibits localization on the plasma membrane [27,38]. Previous studies have revealed that MT1-MMP can perform self-cleavage of its pro domain at the furin cleavage motif [39]. This suggested that MT1-MMP and furin have similar substrates. In the study by Rozanov and colleagues [37], MT1-MMP cleaved both PA83 and PA63 at the cell surface, promoting resistance to anthrax toxin. Thus, while furin cleavage of PA promotes cellular intoxication, MT1-MMP cleavage of PA presumably inhibits this process. It remains to be determined if MT1-MMP cleaves PA in macrophages or other cell types that are implicated during anthrax intoxication [40]. If MT1-MMP cleavage of PA is shown to occur in relevant cell types, then it would suggest that in addition to expression of ANTXRs [41], susceptibility to anthrax toxin is also influenced by expression of MT1-MMP. 

## 9. Conclusions

Studies of the *Antxr1* and *Antxr2* KO mouse phenotypes revealed a critical role for the ANTXR proteins in regulating ECM homeostasis. ANTXRs may facilitate ECM remodeling through their interaction with, and promotion of, MT1-MMP activity. It remains to be determined if the ANTXRs regulate the activity of other MMPs, or other proteases that are known to have a role in ECM remodeling. Does ANTXR primarily target the MT-MMP activation cascade or does the ECM homeostasis defect found in *Antxr* mutant mice include miss-regulation of other proteins involved in ECM biosynthesis and breakdown? These open questions will undoubtedly benefit from the mouse models generated thus far, which have been instrumental in understanding and exploring ANTXRs function in mammals. 
